# Reducing Societal Obesity: Establishing a Separate Exercise Model through Studies of Group Behavior

**DOI:** 10.1155/2016/6734043

**Published:** 2016-06-27

**Authors:** J. S. Puterbaugh

**Affiliations:** Department of Internal Medicine, Providence St. Vincent's Medical Center, Portland, OR 97225, USA

## Abstract

The past 50 years has brought attention to high and increasing levels of human obesity in most of the industrialized world. The medical profession has noticed, has evaluated, and has developed models for studying, preventing, and reversing obesity. The current model prescribes activity in specific quantities such as days, minutes, heart rates, and footfalls. Although decreased levels of activity have come from changes revolving around built environments and social networks, the existing medical model to lower body weights by increasing activity remains individually prescriptive. It is not working. The study of societal obesity precludes the individual and must involve group behavioral studies. Such studies necessitate acquiring separate tools and, therefore, require a significant change in the evaluation and treatment of obesity. Finding groups with common activities and lower levels of obesity would allow the development of new models of land use and encourage active lifestyles through shared interests.

## 1. Introduction

Obesity, defined as a BMI over 30, has been recognized as a health risk since Hippocrates [1, 2], albeit a fairly minor one. That is no longer the case as the National Institute of Health lists obesity and overweight together as the second leading cause of preventable death in the United States, close behind tobacco use [3]. An estimated 375,000 deaths per year in the United States are due to obesity [4] and account for approximately 10% of total US medical costs [5]. Obesity is associated with diabetes, hypertension, strokes, cardiovascular disease, asthma, arthritis, and “poor health status” [6]. Also, it is the nucleus of one of the most deadly medical conditions, the metabolic syndrome or syndrome X first described in 1988 [7]. Obesity is also on the rise in virtually every industrialized area of the world [8].

This remarkable increase in obesity across the developed world reached a historical landmark in 2000 when, for the first time, the number of overweight adults surpassed those who were underweight [9]. Although there has been a perception that obesity seemed to spring suddenly on to the medical horizon in the 1980s [10], recent work has shown that levels of human obesity have been increasing over a much longer period [11].

Historically, the medical profession has been mainly interested in arresting starvation and malnutrition rather than controlling body weight [2]. But when the available food supply matched, and eventually overcame, the energy expenditure of the population, there was a transition period where diseases of insufficiency were gradually supplanted by diseases of excess [2, 11]. The first medical flags were raised about increasing levels of obesity in the 1930s [2, 12, 13]. However, it was in the 1980s that some arbitrary perceptual line was crossed, and obesity became a national and world concern. This is critical as, previously, obesity was perceived as affecting a relatively small number of people whose weight reflected individual lifestyle decisions rather than from issues reflecting societal changes. After about 1980, this perception changed. Obesity became emblematic of changes in the social fabric, of everything from the built environment, jobs, and transportation to the entire food industry.

However, and despite levels of obesity having been on the increase since about 1900 and traceable to technological and societal changes outside the control of individuals, the medical professions continue to treat the problem as an individual's problem.

The current medical model, that is, the model used by most medical practitioners and prescribed for individuals fighting obesity, involves behavioral modification of both diet and exercise. Exercise is generally prescribed numerically in frequency, intensity, and duration as nonfunctional activities, such as a walk or run, or the use of a free-standing exercise facility, a piece of exercise equipment, exercise classes, and videotapes. Thus, the person being prescribed to is told to change his or her lifestyle to allow achievement of these numerical goals.

However, ongoing changes in the built environment, social networks, food supply, food marketing, and eating habits must feed into any systematic evaluation of the levels of obesity in society and more and more seem to be the controlling factors in obesity [12, 15–18].

This paper will review problems of blending these approaches, that is, the evaluation and assessment of changes which have occurred outside of the individual's control, and the individually prescriptive approach, which has been the conventional approach of the medical profession to disease and disability. For clarity, although the changes being discussed are occurring throughout the industrialized world, the United States will be the model country.

## 2. The Evolution of Societal Obesity

What is an acceptable level of obesity in society? Answers to this must be juxtaposed with acceptable levels of malnutrition. For example, the level of obesity in the United States was about 1.5–3.5% in 1900 [19, 20], but malnutrition, as both malnourishment and undernourishment, was rampant [21]. Although societal obesity levels reached some critical mass by the 1980s, the successful elimination of malnourishment which laid the foundation for future obesity began much earlier [11]. The question, therefore, creates a dilemma, as difficulties with the answer relate directly to the difficulties of any solution, so a brief review is needed.

Estimates of the level of obesity in any time frame include three variables: age, period, and cohort effects. Age effects reflect the physiologic and situational (work, parenthood, etc.) changes of life over time. Period effects are the variables which influence all age groups simultaneously and “subsume a complex set of historical events and environmental factors such as world wars, economic crises, famine, epidemics and pandemics of infectious diseases, public health interventions, and technological breakthroughs” [21]. Technological breakthroughs, however, are the only period effects which can create new societal constants and thereby create trend lines. That is, in period studies during times of relatively stable technology, wars, famines, plagues, and so forth cause the cresting and shallows of health variables. But the advent of television or the elimination of polio becomes constants affecting all future cohorts and all ages.

Cohort effects are changes across groups of people usually born the same year or with major life experiences, such as marriage, the same year. Birth cohort studies reflect the combination of both age and period effects (period − age = cohort), as cohorts exposed to a world war or television have different outcomes than those without those experiences.

Evaluating the causes of obesity in times of rapid technological change must rely on cohort studies, as supplying only period information about body weights does not allow access to when body weights were reached. Thus the perception that obesity burst upon the world in the 1980s was based on period studies, whereas cohort studies show that body weights were increasing before the 20th century [9, 11, 15].

**Figure 1:**
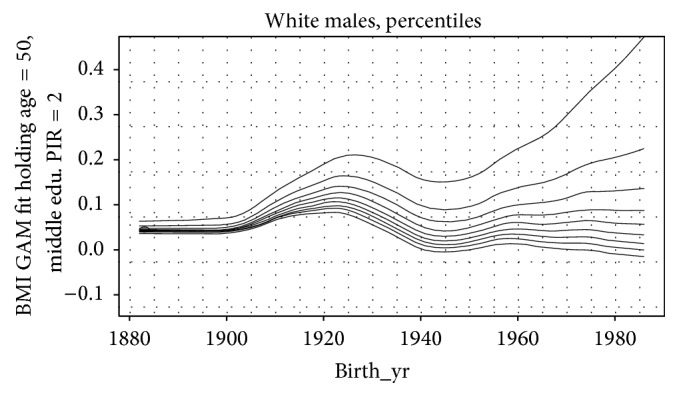
Rate of change of BMI decile curves of white men by birth cohort [14].

These same studies show that weights have increased ever since, with downticks during the war years, because of trend lines established by the accumulation of technological advances (Figure 1).

**Figure 2:**
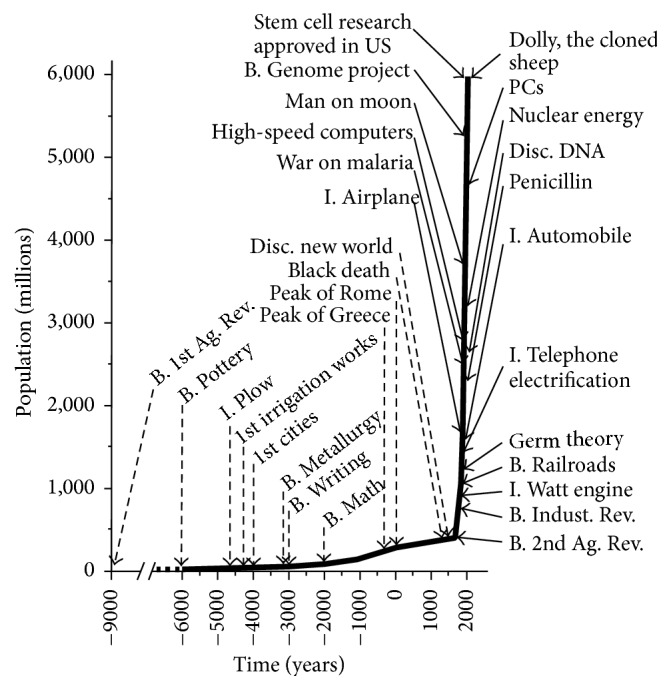
The growth of world population and some major events in the history of technology.* Sources*: Cipolla 1974; Clark 1961; Fagan 1977; McNeill 1971; Piggott 1965; Derry and Williams 1960; Trewartha 1969. See also Allen 1992, 1994; Slicher van Bath 1963; Wrigley 1987 [22, p. 22].

Therefore, technology is the guiding force behind most studies trying to attach causality to increased levels of obesity. The remarkable improvements in the human condition over the past 100 years, gains Fogel grouped as The Technophysio Evolution [22], brought benefits improving virtually every aspect of the human's earthly experience, allowing humans to increase body size by over 50% and longevity by over 100% since 1800. Life expectancy alone increased almost 30 years between 1900 and 1950 [21, pp. 15–40]. The remarkable impact of technology on the human population is best illustrated by Fogel's graph (Figure 2).

**Figure 3:**
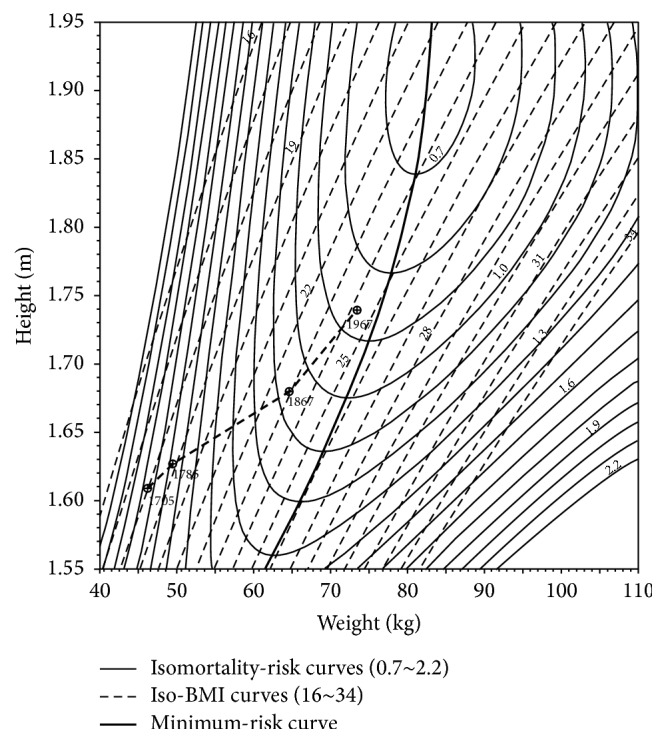
Isomortality curves of relative risk for height and weight among Norwegian males aged 50–64, with a plot of the estimated French height and weight at four dates [22, p. 26].

As lifespan increased, height and weight also did. This relationship is best represented in Waaler's graphs of which Figure 3 is a good example. There is a linear relationship between height and weight in French men at four dates, from 1705 to 1967.

Height and weight are, in turn, closely tied to production of and access to an adequate food supply and income. The production of food has to match demand, and demand has to be consistent with affordability. Nutrition is also aligned with productivity, as well-nourished workers can work harder and longer than undernourished workers but they also require more energy input [22].

It is beyond the scope of this paper to address all the changes required to bring about affordability of the food supply; however, even in the 1930s, only about 50% of the British population was felt to have income sufficient for obtaining an adequate diet [23].

The utility of height and weight in retrograde estimates of nutritional status is weight-limited as height, and thus lifespan at a certain point, plateaus despite increasing body weight. At this point the group under study is becoming obese, and obesity can therefore be defined as a BMI which no longer results in increased longevity [22, pp. 43–66]. Also, increases in height and weight account for virtually all the decrease in mortality from 1705 to 1867, diminishing to about 35% of the decrease after 1867 because of the rapid advances in technology during the ensuing century [22, p. 27, For methods of calculation, pp.113–125]. All data points, however, show increased mortality as BMIs approach 30.

As technology worked wonders at so many levels of human advancement, societies which benefitted never halted their tendency to increased body weights beyond height, productivity, and mortality advantages. These technologically advanced societies simply became heavier, with mortality rates which have levelled off and in some cases even increased [2].

Therefore, cohort studies show the rapid accumulation of changes to the human in the late nineteenth and early twentieth centuries, which included increases in body mass. Malnourishment levels decreased with the understanding of macro- and micronutrients, and, by the end of World War II, as the world healed with improved food production and job production, malnourishment had become a relatively minor problem [2].

Therefore, the answer to the question of when American society was last not obese and not malnourished seems to have been a period lasting 15–20 years after World War II. With peace and adequate food, plus advances in sanitation and disease management, came a significant increase in the level of obesity to about 15% from 1950 to 1970 which, therefore, seems a reasonable goal for a well-fed society, as well-fed is better than underfed. Of some importance, the decrease in activity of the young by the 1950s had already become a national concern [2].

## 3. Developing a New Model for Societal Obesity

It is important to emphasize that all of the given causes for the increased levels of obesity are predicated on societal changes outside the control of the individual. No society has yet managed access to an adequate or superabundance of food and maintained a satisfactory level of obesity. Thus, the current level of obesity in America has climbed to about 35% in adults [24]. The transience of the acceptable societal obesity level is critical as there is no reference period upon which a societal model for the management of obesity can be based. With an adult population of about 250 million, any reasonable attempt to reduce obesity to within even sniffing distance of acceptable levels means affecting major behavioral characteristics of at least 50 million people, something which has no historical precedence. The development of such a model means breaking new ground in the understanding of human conduct in a vast array of demographics.

Goals of any such model for human activity are straightforward. The current level recommended for the human population, based on a cardiovascular model, is 30 minutes of walking equivalent for five or more days a week. Activity required for weight stabilization or loss requires at least 60 minutes of walking equivalence seven days of the week [2]. The percentage of the American population which achieves even the minimal for cardiovascular fitness seems capped at about 20–25 percent of the population [25]. The percentage which meets the requirements for weight stability has not been measured but would be much less. Also, the use of free-standing exercise facilities seems to have levelled off at about 15 million people who use these facilities three or more times a week [26].

Thus, at a minimum, over 80 percent of the population needs to increase activity for both health and weight benefits to accrue. The 50 years of work the medical professions have spent developing the exercise prescription, implementing it, testing and retesting it, evaluating and writing about it in professional and nonprofessional publications, and speaking about it to any listening ear has made no measurable impact on either activity levels or levels of obesity. As technological changes have created new constants for the human which have caused societal obesity, technology could help create the tools necessary to counter it.

The development of a new model for societal obesity has to acknowledge that maintenance of body weight requires over twice the activity level of the cardiovascular model and that this goal is unlikely to be met without societal changes which return exercise to a secondary rather than a primary goal. More recondite factors, such as urban sprawl, occupation, neighborhood walkability, and societal networks, require better understanding in order to clarify causality from correlation [12, 16, 17, 27, 28].

Such a model must put observations before theory with the profession actively engaging in the development of a societal prescription. This was recognized as early as 1930 by a physician commenting on the medical profession advising changes in behavior: “Men are not impelled to action by intelligence one tenth as powerfully as by emotion…A careful study must be made of human incentives and motives. Without these, the health prescription falls of its own weight and no good has been done” [29]. The analytic and prescriptive methodology for understanding and controlling disease simply does not work for problems related to human behavior.

An observational model would encourage research to extend beyond individual behavior and include studies of people sharing common activities who achieve lower levels of obesity. These “established success groups” would change the paradigm from individuals to groups. For example, woodworkers seem to have a lower level of obesity [30], as do gardeners [31] and golfers who walk the course [32]. Studies of bird watchers, beekeepers, backpackers, dancers, skiers, and so forth would help break ground on the establishment of a societal model based on proven success. This would also return the perception of human activity to the productive and enjoyable rather than the nonproductive and boring characteristics of the current prescriptive model. Group behavioral studies would also encourage better land use to allow these interests and activities to flourish [33].

## 4. Conclusions

Developing innovative analytic tools and an existential activity model is critical to the control of societal obesity. This model should be based on studies of group behavior, societal demographics, and altering the built environment to encourage active lifestyles.
